# The generation mechanism underlying the career decision-making difficulties faced by undergraduates in China during the COVID-19 pandemic: a qualitative study based on SCCT theory

**DOI:** 10.3389/fpsyg.2023.1154243

**Published:** 2023-06-12

**Authors:** Hairong Shi

**Affiliations:** School of Biology and Food Engineering, Chuzhou University, Chuzhou, China

**Keywords:** career decision-making difficulties, undergraduates exhibiting delayed employment in China, COVID-19, SCCT theory, career self-management model, mind sponge theory

## Abstract

As COVID-19 continues to spread worldwide, the record number of graduates in China and pressure resulting from the economic downturn have led to low confidence in employment among college students, and the difficulties associated with career decision-making have gradually developed into a psychological barrier to the successful employment of Chinese college students. Using the “purposive sampling” approach to qualitative research, this study selected 20 undergraduates exhibiting delayed employment from a university as our research sample and used the career self-management model of social cognitive career theory (SCCT) as an analytical framework to conduct semistructured interviews with the aim of exploring influencing factors associated with and generation mechanism underlying the career decision-making difficulties experienced by Chinese undergraduates during the COVID-19 pandemic. According to the career self-management model of SCCT theory, the four variables of individual, parents, peers and social environment influence Chinese undergraduates’ career decision-making difficulties. On this basis, this study proposes a multivariable and single-subject generation mechanism to explain undergraduates’ career decision-making difficulties and tries to explicate the mental changes associated with the career decision-making difficulties encountered by undergraduates exhibiting delayed employment by reference to mind sponge theory.

## 1. Introduction

2022 was the third year of the global spread of the COVID-19 epidemic; during this time, mutant strains of the virus emerged in rapid succession, the global economic situation was grim, and the economic growth rate was slow. Due to the continuous impact of the epidemic, China’s economy was also severely impacted. In response to unfavorable forecasts regarding the future market environment, many small and medium enterprises reduced cost pressures through layoffs and lowered recruitment quotas, which greatly decreased employment demand. On the other hand, data provided by the Ministry of Education showed that the number of university graduates increased year by year, with 10.76 million graduates in 2022, an increase of 1.67 million over the same period the previous year; in addition, both the scale and increment of such graduates had reached a record high (Ministry of Education, 2021). Therefore, the employment of college graduates was affected not only by the record number of graduates and the downward pressure of the domestic economy but also by the sudden, cumulative, and superimposed impact of the COVID-19 epidemic. The employment stability of college graduates decreased, unemployment worries increased, job satisfaction declined significantly, and the proportion of students entering higher education increased significantly. The proportion of college students entering nonpublic enterprises also decreased significantly, while the proportion of college students employed in the entry system increased slightly (Liu et al., 2022). The COVID-19 epidemic had a tremendous negative impact on the employment of Chinese college graduates. Thus, graduates tended to change their places of employment and adjust their expected salaries and anticipated industries; furthermore, the proportion of graduates choosing “delayed employment” increased, while the proportion of graduates receiving offers and actually signing contracts decreased significantly (Mao et al., 2022). During the COVID-19 pandemic, college students’ employment cognition exhibited “low self-efficacy”; 72.2% of college students agreed that “the employment situation over the next 2 years would be very severe,” and only 20.9% of college students agreed that “I believed I could find a job order when I graduated. Only 29.9% of college students agreed with the statement that “I had a clear plan for my career,” college students’ confidence in their ability to obtain employment was low, and they exhibited great uncertainty with regard to their personal development (Li et al., 2021). Individuals experienced “entanglement,” “anxiety” and “confusion” in the context of career decision-making, which became a common complaint on the part of college students in the postepidemic era. The difficulty of career decision-making gradually developed into a psychological barrier to Chinese college students’ employment and became an obstacle and constraint with regard to their successful employment. Therefore, this study posed the following research question: During the COVID-19 pandemic, what factors influenced college students’ career decision-making difficulties? On the basis of this question, this paper comprehensively analyzed the manner in which career decision-making difficulties were generated.

## 2. Literature review

The notion of career decision-making was first proposed by British economist Keynes and other researchers. The career decision-making is a complex cognitive process that decision-makers can use to organize information about themselves and their career environment, carefully consider the various career prospects available to them and make a public commitment to certain career behaviors (Jepsen, 1984). Career decision difficulty is a relatively new concept in the field of occupational psychology. The notion of career decision difficulty refers to an individual’s inability to select or commit to a course of action with regard to preparing for or entering a particular career (Crites, 1969).The essence of career decision-making difficulties lies in dissatisfaction with decision-making, by a decision-making state caused by insufficient career-related learning experience or by the fact that the individual has not learned or applied a systematic approach to career decision-making. These difficulties are natural consequences of learning experiences (Krumboltz, 1993). The career decision-making difficulties include the various difficulties that individuals may encounter during the process of making career decisions (Gati et al., 1996). The difficulties associated with career decision-making emerge when individuals do not know what career they ultimately want to pursue or which of several careers they should choose in the process of career choice (Long and Peng, 2000); it is not merely an obstacle to career decision-making but also represents an individual’s response to such an obstacle, which is diffused in the process of career decision-making (Liu and Zhang, 2021). In summary, career decision-making difficulties focus on decision-makers’ inability to make decisions, their dissatisfaction with decision-making results, the difficulties they encounter in decision-making, and their reactions to those difficulties. The difficulty of college students’ career decision-making has received increasing attention due to increasing employment pressure in China.

Empirical studies have shown that an individual’s career decision-making style, personality traits, ability, self-efficacy and other factors affect career decision-making difficulties. An adaptive career decision-making style directly reduces adolescents’ career decision-making difficulties, improves adolescents’ self-efficacy, and promotes positive changes in their career decision-making status (Gadassi et al., 2013). Among the Big Five personality traits, initiative is significantly negatively correlated with career decision-making difficulties, and ability is significantly negatively correlated with career decision-making difficulties (Taylor, 1982). Self-efficacy has a significant negative predictive effect on the extent of career decision-making difficulties (Taylor and Betz, 1983; Nota et al., 2007). In addition, individual differences in gender, age, major and grade also affect career decision-making difficulties. Career decision-making difficulties decrease with increasing age or grade; Liberal arts students are more prepared than science students; senior students are more strategic with regard to collecting information than junior students; and male students exhibit significantly higher internal conflicts, external conflicts and unreasonable beliefs than female students (Gati et al., 1996; Gati and Saka, 2001). Family environment factors are significantly correlated with the dimensions of lack of preparation, difficulty in exploring information and conflict and could predict the dimensions of career decision-making difficulties among college students (Zhao, 2013); family attachment style, psychological separation and parenting style affect career decision-making difficulties (Darling and Steinberg, 1993; Tokar et al., 2002). Prior to the outbreak of the novel coronavirus pneumonia epidemic, research results indicated that the career decision-making difficulties faced by Chinese college students were at a medium level and that the various dimensions of career decision-making difficulties exhibited different characteristics. The order of these factors was social environment factors > occupational familiarity > self-evaluation ability > social adaptability > person-job matching; simultaneously, the personality, career values, career decision-making effectiveness, decision-making style, social support, and family parenting style of college students all affect the difficulties they encounter in their career decision-making (Chen, 2014). In the context of the global COVID-19 pandemic, Chinese college students exhibit a greater preference for job stability and risk resistance, fully consider the impact of emergencies on their career decision-making, and pay more attention to job stability, fixity, safety, health, challenge and risk (Ye et al., 2022).

## 3. Theoretical basis: the career self-management model of social cognitive career theory (SCCT)

SCCT is a social cognitive theoretical framework for careers, and this theory was developed by Lent and others based on Bandura’s social cognitive theory (Lent et al., 1994; Brown and Lent, 1996). The three core variables included in the theory are self-efficacy, outcome expectations, and personal goals, and it emphasizes the systematic integration of individual psychology, social background, economic development, and other factors and thus the “individual-behavior-environment” interaction (Lent and Brown, 2006a,b). SCCT consists of four interrelated models, i.e., the career interest model, the career choice model, the performance model, and the job satisfaction model, which dynamically explicate the formation of career interest, the process of career choice, the acquisition of career performance, and the impact of work-life satisfaction in this context (Sheu and Bordon, 2016; Lent and Brown, 2019). Based on these four models, Brown and Lent proposed a fifth theoretical model: the career self-management model. This model explains people’s occupational adaptive behavior in the process of career development. It maintains that self-efficacy and outcome expectations affect career exploration and that self-efficacy, outcome expectations, and career exploration lead to the final career outcome. In the process of individual career decision-making, self-efficacy, outcome expectations, goal-setting, environmental factors, and individual personality are interrelated, and they jointly determine the outcome of career decision-making (Lent et al., 2013). The self-management model focuses on the process of adaptive occupational behavior (such as career exploration, job search, career development) based on career path, which involves job or career changes and emphasizes personal and environmental variables, such that the study of individual career change focuses on self-efficacy, outcome expectations, goals, supports and obstacles, and related personal characteristics. These factors are interrelated and jointly determine the persistence of adaptive exploration and decision-making behavior, and they cannot operate as separate influences (Brown and Lent, 2019). This model is applied to career exploration and decision-making behavior. Environmental variables, as important factors, operate via multiple pathways, directly promote goals and actions, moderately strengthen or weaken the relationship between goals and actions, strengthen self-efficacy and outcome expectations, and directly affect adaptive behavior (Lent et al., 2013; Brown and Lent, 2019). In this study, Chinese college students’ career decision-making difficulty refers to a kind of career adaptive behavior disorder that occurs during the process of individual career decision-making. The COVID-19 pandemic has caused Chinese college students to face many uncertain external obstacles with regard to their career decision-making behavior. The self-management model of SCCT theory thus provides a very appropriate theoretical framework for the analysis conducted in this study.

## 4. Research design

### 4.1. Research methods and questions

To date, most relevant studies conducted both at home and abroad have used quantitative research methods, such as descriptive statistics, correlation analysis and regression analysis, to analyze the relevant data. The static cross-sectional data included in the sample face certain limitations with regard to explaining the corresponding dynamic relationships among the variables, which made it difficult to explore the generation mechanism underlying the deep-seated influencing factors of the sample with regard to career decision-making difficulties in further detail (Koumoundourou et al., 2011; Chen et al., 2012; Slaten and Baskin, 2014; Kim et al., 2016; Karacan-Ozdemir, 2019; Chuang et al., 2020; He et al., 2021; Parola and Marcionetti, 2021; Jia et al., 2022). Qualitative research provides an “explanatory understanding” of the subject’s personal experience and meaning construction based on face-to-face communication between the researcher and the subject. The researcher explains the subject’s life story and meaning construction by reference to his own experience. The researcher himself operates a research tool used to study the individual’s “life world” in a natural situation (Chen, 2000). As a method of qualitative research, the case study can be applied in three situations: in the first situation, the main problem pertains to “how” and “why”; in the second, the researcher can hardly control the research object; and in the third, the focus of the study is the current reality (Yin, 2014). Therefore, this study was a qualitative study that took a Chinese university undergraduate as a case and used the case study method to interpret the influencing factors and generation mechanism associated with undergraduate career decision-making difficulties.

### 4.2. Case selection

Purposive sampling uses limited resources to gather the most effective information, such as by identifying and selecting informative individuals or groups of participants relevant to the phenomenon of interest (Palinkas et al., 2015). According to the principle of “purposive sampling,” this study extracted research objects that could provide the maximum amount of information and focused on research objects that could provide intensive and rich information for the research topic when used as cases (Levitt et al., 2018). For various reasons, college students exhibiting delayed employment have extended the time they take for the process of job selection, resulting in a long period of time during which they are unable to participate in relatively stable formal work, hesitation in making various employment decisions, difficulty in making final decisions, and varying degrees of career decision-making difficulties (Zheng and Wang, 2019). Therefore, this study recruited undergraduates exhibiting delayed employment as a case study from a university. The recruitment criteria were as follows: 1. 2022 undergraduate graduates; 2. Not formally employed at the time of graduation in July 2022; 3. Voluntary participation in this study and sufficient language skills to overcome barriers to communication; 4. Willing to exchange employment topics and skilled at expressing their thoughts. Recruitment information was published through the researcher’s network, including recruitment criteria, the purpose of the research, data confidentiality, and the right to withdraw at any time without providing a reason. According to the recruitment information, 36 research cases were selected from 153 people according to the three criteria of gender balance, professional balance, and self-description. The materials were collected through semistructured interviews, and the interview order iterated between male and female participants. When the 20th case was interviewed, the data collection was repeated following the same approach as used previously, and no new data emerged; thus, the data collection had reached saturation. Ultimately, 20 cases were interviewed, all of whom volunteered to participate in the study, and no participants ceased to participate at the midpoint of the research. The sample included 10 male students (indicated by the letter A) and 10 female students (represented by number B); 11 students majored in the natural sciences, while 9 students majored in the humanities and social sciences. To ensure the privacy of the interviewees and the confidentiality of the data, code names are used instead of real names in the full text (see Table 1).

**Table 1:** Basic information of the interviewees.

Number	Gender	Discipline	Number	Gender	Discipline
A01	Male	Natural Sciences	B01	Female	Humanities and Social Sciences
A02	Male	Humanities and Social Sciences	B02	Female	Natural Sciences
A03	Male	Natural Sciences	B03	Female	Humanities and Social Sciences
A04	Male	Natural Sciences	B04	Female	Natural Sciences
A05	Male	Natural Sciences	B05	Female	Humanities and Social Sciences
A06	Male	Natural Sciences	B06	Female	Humanities and Social Sciences
A07	Male	Humanities and Social Sciences	B07	Female	Natural Sciences
A08	Male	Natural Sciences	B08	Female	Humanities and Social Sciences
A09	Male	Natural Sciences	B09	Female	Humanities and Social Sciences
A10	Male	Humanities and Social Sciences	B10	Female	Natural Sciences

### 4.3. Data collection

From July to September 2022, this study used semistructured interviews to collect first-hand data; this process mainly involved face-to-face interviews, which were supplemented by online video interviews. Fourteen people participated in face-to-face interviews, and 6 people participated in online video interviews. Before the interviews, a structured interview outline was developed to guide the researcher during data collection. The interview outline consisted of three open-ended questions: 1. During COVID-19, did you encounter any problems in your career choice process? 2. What personal factors did you think constrained employment choices during COVID-19? 3. What external factors did you think affected employment choices during COVID-19? Following the interview outline, the researcher encouraged the interviewees to participate actively and ask questions, and the researcher flexibly adjusted the corresponding content and procedures to suit the interview situation at hand. Interviewees freely shared their experiences of employment distress and reflected on the factors that influenced their employment choices until both sides fully understood the emerging themes. The researcher had extensive experience with qualitative interviews and asked more detailed and in-depth follow-up questions when necessary. When newly collected data and previously collected data exhibited the same content and no new data emerged, the data were considered to have reached saturation, and the data collection was completed. The entirety of the interviews were recorded with the consent of the interviewees before the interviews, and each interview lasted 42–65 min.

### 4.4. Data analysis

After the interview, the recording was transcribed by the researcher word by word, and the preliminary data collation was completed, which provided direction and focus for subsequent data collection. In this study, the computer software NVivo 11 was used to log and code the original data, and the generic analysis method was used to identify the common themes of the data. The self-management model of SCCT theory was used as the analytical framework, and the spiral research model was used to classify, analyze and organize the data systematically. Data analysis was conducted simultaneously by three staff members. When these staff members expressed inconsistent opinions regarding the analysis, they engaged in discussion until a consensus was reached. Transcribed data, extracted themes, and sub-themes were fed back to the interviewees, who were contacted once again after obtaining their consent. After repeated communication and feedback, this process ensured that the results of the study did not deviate from the perspective of the researchers and could accurately reflect the views of the interviewees.

## 5. Results

According to the career self-management model of SCCT theory, the career decision-making difficulties faced by undergraduates were the result of the joint influence of individual variables and environmental variables. This study found that among all the relevant variables, the individual variables included Chinese undergraduates’ career decision-making self-efficacy, outcome expectations, goal-setting and individual personality; in turn, the environmental variables included not only important others in the process of career decision-making, such as parents, but also strong peer relationship groups, such as groups of roommates, lovers, friends, and classmates. This category also included external, objective factors, such as the background of the COVID-19 pandemic. This study used NVivo 11 software to log and code the interview text data. The self-management model of SCCT theory was used to analyze the influencing factors of career decision-making difficulties indicated by interviewees, which were summarized into two nodes, i.e., individual factors and environmental factors, and seven child node, i.e., low career self-efficacy, negative career outcome expectations, career goal dilemmas, limitations pertaining to individual personality factors, parental influence, peer influence, and social environmental influence. The specific coding is shown in Table 2.

**Table 2:** Types of and influences on the career decision-making difficulties experienced by college students exhibiting delayed employment.

Node	Child node	Number of sources of materials	Number of reference points	Coding example
Individual factor	Career self-efficacy is not high	Thirteen	Twenty-five	My major is relatively unpopular; and my school is also very ordinary. I personally do not have particularly outstanding ability, and now it is very difficult to find any job; every day is very anxious, and the pressure is relatively high.
	Negative expectations regarding career outcomes	Fourteen	Twenty-nine	It’s too difficult to find a satisfactory job now. I’m looking forward to working closer to home and taking care of my family. It does not matter whether my income is high or low.
	Career goal dilemmas	Nine	Eighteen	I do not have a clear plan for my career development, and I do not know how to set up my future career goals step by step and find a job to solve the problem of survival first.
	Limitations pertaining to individual personality factors	Seven	Thirteen	My senior job search is relatively smooth, job hunting is more proactive, and I have received input notices from four enterprises input. Each of the four units has its own merits and demerits. I repeatedly rejected two units. The remaining two units made me too entangled. I missed the employment opportunity due to hesitation and graduated in a flash. I regretted it.
Environment factor	Parental influence	Four	Six	When I was young, my parents were strict with me. I signed up for many interesting classes. Everything had to be arranged by my parents. I seldom had the chance to make my own decisions. I did not know what to do when I was looking for a job.
	Peer influence	Fifteen	Thirty-three	Under the influence of my roommate, I joined their team and indulged in the joys and sorrows of the game. As a result, I neglected my studies. I think it’s really hard to find a job now.
	Social and environmental impacts	Nineteen	thirty-nine	During the epidemic, when an enterprise posted a job on the internet, at least a dozen people sent resumes, and more than a hundred people sent resumes, which was too difficult for introverts.

### 5.1. Individual variables

#### 5.1.1. Professional self-efficacy is not high

Self-efficacy is a key psychological factor according to SCCT theory. This term refers to the individual’s personal beliefs regarding his or her ability to perform specific actions or engage in specific behavioral processes (Lent et al., 2013). Self-efficacy can be assessed in terms of task-specific self-efficacy, process efficacy, and obstacle-specific coping efficacy (Lent and Brown, 2006b). In this study, the career decision-making self-efficacy of undergraduates exhibiting delayed employment was found not to be high overall; most such undergraduates lacked confidence in their career decision-making, underestimated their decision-making skills, and wavered excessively when faced with a variety of choices, believing that they could not overcome decision-making anxiety: *“My major is relatively unpopular, and my school is very ordinary, I do not have particularly outstanding ability, and now it is very difficult to find any job; every day is very anxious, and the pressure is relatively high” (A7); “In the next semester of my junior year, I am going to apply for graduate students. I bought materials and have been preparing for the exam. But in the first semester of my senior year, I saw my classmates find jobs in enterprises. Soon after I went to work, I received my first month’s salary. I felt very good. I wanted to go to work. I had been preparing to become a graduate student for a long time. It was a pity to give up. I was particularly entangled and contradictory. The whole experience made me very anxious” (B10).*

#### 5.1.2. Negative expectations regarding occupational outcomes

Outcome expectations refer to beliefs regarding the outcome of performing a particular action or taking a particular course of action (Lent et al., 2013). Bandura (1986) focused on three types of intended consequences: social (family benefits), material (economic gains), and self-evaluation (self-recognition) outcomes The self-efficacy associated with career decision-making produces valuable outcome expectations with regard to participation in career decision-making activities, which encourages individuals to participate in career decision-making activities continuously (Lent et al., 2013). Based on the results of this study, in accordance with Bandura’s classification of expected outcomes, the career outcomes of undergraduates exhibiting delayed employment were generally negative; most such undergraduates were not optimistic with regard to their ability to find the job they desired, and it was difficult for them to confirm that their job would provide satisfactory family benefits, objective economic benefits and a high degree of self-recognition and other material or spiritual gains: *“It’s too difficult to find a satisfactory job now. I’m looking forward to working closer to home and taking care of my family. It does not matter whether my income is high or low. I’m trying to find one now, and I do not know what the result will be” (B4); “The economic situation of my family is good; my family supports my development and interests, and I pay more attention to harvest and growth in work, gaining a self-identity and exhibiting self-improvement; space for development and work potential are more important to me. I do not know when I can find it, so I can only look for it” (A6).*

#### 5.1.3. Career goal dilemmas

Personal goals refer to the intention to engage in a specific activity or specific field as well as the intention to achieve a specific level of task performance and a specific outcome (Lent et al., 1994, 2002). During the current precarious times, which are characterized by the COVID-19 pandemic, it can also be very challenging for individuals to set goals. Goal setting is a powerful strategy for behavioral modification. Setting clear and difficult goals and providing relevant feedback can improve task performance. Goal setting includes five important components: clarity, challenge, commitment, feedback, and task complexity (Locke and Latham, 2006; Konstantara and Galanakis, 2022). According to the results of this study, undergraduates exhibiting delayed employment faced prominent problems when setting career goals, such as low clarity, the lack of a challenge, and the lack of immediate feedback regarding their goals: *“I do not have a clear plan for my career development, and I do not know how to set my future career goals. I take one step at a time and find a job to solve the problem of survival first” (A3); “It is not easy to survive during the epidemic. All walks of life are relatively introverted. I just want to find a stable and comfortable job. I do not want too much pressure. Too competitive career goals are not suitable for me” (B8); “My goal has been relatively clear. I hope to be admitted to the civil service. During my senior year, I have been preparing for the exam and have participated in various national, provincial, and municipal examinations. The results of the civil service examination are relatively slow; you wait for one or 2 months, the initial score is in line with the re-examination score line, you have a second round of interviews, and then you wait for the results, which takes a long time in the middle, and feedback regarding the civil service admission results is particularly slow. I took five exams in my senior year, but I was not admitted. I am going to take the exam for another year” (A8).*

#### 5.1.4. Limitations pertaining to individual personality factors

The proactive personality significantly and negatively predicts college students’ career decision-making difficulties (He et al., 2021). “The traits of the five-factor model of personality can predict difficulties in career decision-making, There was a statistically significant positive relationship between Neuroticism and difficulties in career decision-making, and statistically significant negative relationships between Agreeableness, Conscientiousness, Extraversion, and Openness” (Martincin and Stead, 2015). Therefore, this study proposes that personality traits are core factors in the career decision-making process. The mixed perfectionism sub-types as entailing a high risk with regard to career decision-making, while non-perfectionism sub-types were associated with low career decision-making difficulties (Wang et al., 2020). College students pursued “perfection” to a greater or lesser degree and exhibited a higher level of career decision-making difficulties. The perfectionism of college students was significantly related to their career decision-making difficulties; negative perfectionism led to career decision-making difficulties, and positive perfectionism could reduce career decision-making difficulties (Chen H. et al., 2022). “Indecisive” individuals usually exhibited habitual indecision in the context of decision-making (Gati et al., 2011; Zhang et al., 2016). Personality factors might influence occupational adjustments by promoting (or preventing) behavioral performance or by encouraging emotional coping tendencies (Lent et al., 2013). This study found that the personality traits exhibited by undergraduates exhibiting delayed employment included not only perfectionism but also indecision, depression, and neuroticism: *“My senior job search is relatively smooth, the job hunting is more proactive; I have received offers from four enterprises. Each of these four enterprises has its own merits and weaknesses. I repeatedly compared and rejected two units, and the remaining two units cause me to become too entangled. I missed the employment opportunity due to hesitation and graduated in a flash. I think about it and regret it” (B3).*

### 5.2. Environmental variables

#### 5.2.1. Parental influence

Parents’ adoption of an emotional, warm, and understanding parenting style could encourage their children to be more proactive in their careers and learning and ensure that those children encounter fewer difficulties in career decision-making (Zhao, 2012). In the regression analysis of the dimensions of career decision-making difficulties, The career decision-making difficulties faced by college graduates were related to parental rearing patterns. The support and encouragement of parents caused college students to experience more social support, and the degree of difficulty they faced in the context of career decision-making was relatively low; children who grew up in families that emphasized severe punishment and excessive interference from their parents exhibited more uncertainty in their career decision-making, and the decreased social support they received caused them to fail to engage in positive career exploration and to lack self-confidence; accordingly, they encountered greater difficulties in their career decision-making (Lv, 2010). Parental interference and lack of engagement had positive direct effects on career decision-making difficulties (Parola and Marcionetti, 2021). According to the results of this study, the influence of parents on unemployed college students was very obvious. Most students experienced a very strict family education. The high expectations, high standards, and high control exhibited by their parents made it difficult for such students to cope with the uncertainty associated with their career decision-making calmly. They were not confident, wavered, and were hesitant in the face of career decision-making: *“When I was young, my parents were strict with me. I signed up for many interesting classes. Everything had to be arranged by my parents. I seldom had the chance to make my own decisions. I did not know what to do when I was looking for a job” (A6).*

#### 5.2.2. Peer influence

A representative definition of peer influence is as follows: “peer influence was defined as instances where one person affected or was affected by, one other or multiple others who were similar in age. Compelling evidence demonstrates that peer influence was a pervasive force during adolescence, one that shaped adaptive and maladaptive attitudes and behaviors” (Laursen and Veenstra, 2021). Such influence causes people to experience a sense of admiration, dependence, and intimacy and the surrounding groups to engage in a sincere and conscious imitation of their psychology and behavior; it is associated with the characteristics of equality, metaphor, and authenticity (Chen and Lu, 2021). “Adolescence was a period of rapid cognitive, social, and physical transformation. The form, pace, and scope of these changes increase the perceived need for similarity with peers, leaving adolescents vulnerable to peer influence. This study advanced the influence-compatibility model, which argued that peer influence serves to increase similarity with friends and peer group affiliates, which in turn promotes compatibility. The cultivation of compatibility was essential for success in the adolescent peer world because it made one a more desirable companion and reduced the risk of friendlessness and exclusion.” (Laursen and Veenstra, 2021). Therefore, harmonious peer relationships play an important role in the growth and development of contemporary college students and represent the main source of experience that enables these students to enter society and the workplace in the future. According to the results of this study, the most influential peers of unemployed college students were roommates and lovers: *“There are six people in the dormitory, including four roommates who like playing games very much. I do not like playing games very much. They often play online games in groups. When I go back to the dormitory, I watch them shouting for the game every day. They are happy like a child for a while, and the keyboard is broken for a while. I am very curious about how the game has so much charm. Under the influence of my roommates, I also joined their team, indulging in the joys and sorrows of the game. As a result, my studies were neglected. I think it is really difficult to find a job now” (A10); “I have a boyfriend who was studying for the same major in college. He is a straight student with an excellent character, and he learns well. He wants to go to a first-tier big city to study as a graduate student and to get married there after graduation. He hopes that I can work hard with him. I am a Buddhist who pursues a comfortable life. Life in a big city is tense and stressful. I do not want to work and live in a first-tier city. Throughout my senior year, I struggled with whether to go back to my hometown to find a comfortable job or to follow my boyfriend to fight? I also missed out on some good job opportunities while wasting time” (B8).*

#### 5.2.3. The social environment against the backdrop of the COVID-19 pandemic

In China, the COVID-19 epidemic was basically controlled effectively in 2022, but the disease continued to spread in some regions and continues to affect economic growth and people’s lives in some regions to a substantial degree. The Chinese economy faced great uncertainties with respect to the development of the international economic situation and the growth of external demand. Chen and others predicted that the economic trend would exhibit steady development throughout the year. This trend of steady progress was slightly lower before the pandemic and slightly higher afterward (Chen H. et al., 2022; Chen X. et al., 2022). Based on a sample survey of 34 colleges and universities in 19 provinces, Yue and others found that the overall employment rate of college graduates in 2021 was 76.5%, which was 3.6 percentage points lower than the rate in 2019, i.e., prior to the epidemic; in addition, the proportions of unit employment, flexible employment and travel abroad decreased (Yue and Qiu, 2022). The COVID-19 epidemic had many negative impacts on the employment of new graduates: job interviews were prevented, the employment rate declined, employment pressure increased, and economic expectations of the future tended to be pessimistic (Li, 2020). The COVID-19 epidemic reduced employment opportunities and limited geographical circulation in the Chinese labor market. On the one hand, enterprise recruitment and employment demand became more cautious during the epidemic. Many enterprises suspended or reduced labor recruitment, and graduates faced conditions of unprecedented panic and anxiety; on the other hand, the regional mobility partition caused by the postepidemic caused college students to engage in deep thinking regarding their choice of career regions (Zhou, 2022). According to the results of this study, the impact of the COVID-19 epidemic on the career decision-making difficulties faced by unemployed college students focused on two aspects: the serious negative mental effects associated with career decision-making, such as depression, job-hunting anxiety, unemployment panic, and helplessness in choosing jobs, on the one hand, and increasing external obstacles to self-employment, such as regional barriers to epidemic prevention and control, fierce employment competition and reduced employment opportunities, on the other hand: *“I was depressed throughout my senior year, and it was not very easy to find a job. I sent my resume to nearly 50 companies, some of which sent interview notices, and most of which required online interviews. I interviewed at school, and the environmental conditions were not very good. During the epidemic, a company posted a job opening online. At least a dozen people submitted their resumes, and as many as hundreds of people submitted their resumes. The competition was too fierce. I was facing the harsh reality of ‘unemployment upon graduation’, and I was under a lot of pressure to survive. I am very anxious about my unemployed status and hope to find a job in a hurry to support myself” (B2).*

### 5.3. Single subject and multiple variables: the generation mechanism of career decision-making difficulties

With regard to all the variables, the individual was the only subject and ultimate producer of career decision-making difficulties, parents were the most substantial environmental variables affecting career decision-making difficulties, peers were the surrounding environmental variables associated with career decision-making problems, and the social environment was the peripheral environmental variable pertaining to career decision-making problems. This study proposed that a generation mechanism consisting of “single subject and multiple variables” –was associated with career decision-making difficulties. With regard to this mechanism, three pairs of variables affected each other. The first such relationship was the mutual influence between career decision-making difficulties and individuals. On the one hand, the generation of individual career decision-making difficulties was caused by the lack of individual career motivation, and low self-efficacy with regard to career decision-making led to negative expectations of the results of career decision-making. Both factors led to ambiguity with respect to the objectives of career decision-making and subsequently to the emergence of behavioral barriers to career decision-making. Simultaneously, the limitations of personality factors could also cause ambiguity with regard to career decision-making goals and behavioral barriers; on the other hand, the behavior associated with career decision-making difficulties could inhibit the individual’s sense of professional efficacy and cause the individual’s career motivation to be insufficient. The second such relationship was that career decision-making difficulties and the environment shape each other. Parents, peers and the social environment were identified as the external forces associated with career decision-making difficulties, which were surrounding environmental factors and could lead directly to the emergence of individual career decision-making difficulties. Simultaneously, individual career decision-making difficulties also affected parents and peers as environmental variables and allowed these two types of environmental variables to be reconstructed. The third such relationship was the interaction between the individual and the environment. The external environment was gradually internalized by the individual as the individual’s career decision-making self-efficacy became inhibited, thus affecting the individual’s “internal drive”; this change in the individual also subtly infected and affected the individual’s parents and peers as environmental variables.

## 6. Discussion

### 6.1. Mind sponge: a new perspective on the generation of career decision-making difficulties

Although this study explains the generation mechanism underlying undergraduates’ career decision-making difficulties with the help of the career self-management model of SCCT theory and dynamically explicates the whole process of career choice and the relationships among related variables, it cannot explain the mental changes that occur in the career decision-making difficulties encountered by Chinese undergraduates over time in further detail. The notion of a mind sponge is a metaphor according to which the mind resembles a sponge, absorbing new compatible values and squeezing out values that are incompatible with its own core values, comfort zones, multifiltering systems, information and environment. The notion of a core value is used to judge the newly absorbed value (or information) and to make corresponding decisions or responses, thereby creating a self-protection mechanism for the individual “self”; the comfort zone refers to the context in which the multifiltering system evaluates the appropriateness and usefulness of newly input values, thereby helping protect core values from external shocks; the multifiltering system has the two basic functions of information integration and discrimination and is driven by three-dimensional filters, inductive attitudes and trust evaluators; finally, information can be absorbed by the mind only if it is available and accessible. Information passes in and out in an uninterrupted, continuous flow, thus highlighting the capability of the mind sponge for renewal (Vuong and Napier, 2015; Nguyen et al., 2022). The notion of the mind sponge is helpful when studying psychological phenomena in terms of their temporal dimension with regard to the information process associated with the natural renewal of human psychology and society, which can explain and help address complex psychological and behavioral problems (Nguyen et al., 2022). Therefore, this study attempts to use the notion of the mind sponge to explain the mental changes that occur in Chinese undergraduates exhibiting career decision-making difficulties over time. In the process of career decision-making, the individual’s core values are relatively stable individual personality and career-related values, which represent the core part of the individual’s “self-image”; individual career decision-making self-efficacy, outcome expectations and goal setting are located in the individual’s personal comfort zone, which protects the individual’s core values from the direct impact of the external environment; in addition, the individual’s parents, companions and social environment, as the external environment surrounding the independent individual, are composed of constantly changing environmental factors and are sources of individual information. The difficult employment situation, negative peer influences and strict parental discipline associated with the epidemic, as continuous new stimuli in the environment, constantly act on the comfort zone and core value zone of Chinese undergraduates via the screening of the multifilter system, leading to low self-efficacy, negative outcome expectations and obstacles to goal setting in the process of employment. Negative employment experience gradually leads to the formation of a mentality of difficult career decision-making on the part of undergraduates exhibiting delayed employment.

### 6.2. Intervention strategies for the career decision-making difficulties experienced by college students exhibiting delayed employment

Based on the factors influencing the career decision-making difficulties of college students exhibiting delayed employment, this study proposes that alleviating the career decision-making difficulties of college students exhibiting delayed employment must begin with the following four aspects. First, it is suggested that college students with delayed employment should constantly strengthen their career planning, establish reasonable career expectations, continually improve their professional abilities, develop optimistic attitudes and positive thinking modes, constantly improve their career self-efficacy, gradually change their career outcome expectations from negative to positive, break through the dilemma of career goals and improve their personality-based limitations. Second, it is suggested that the family support of college students exhibiting delayed employment should be strengthened. Parents should give college students exhibiting delayed employment more space and opportunities to choose themselves, encourage them to develop confidence in their career choice, and help them overcome the dilemma of career decision-making difficulties as soon as possible by providing warm affection and selfless care. Third, it is suggested that colleges and universities should continue to pay attention to the job-hunting situation faced by college students exhibiting delayed employment, help them adapt to the workplace as soon as possible by providing employment information, employment skills training, and career planning guidance as well as encouraging career concept change; in addition, these institutions should help such students complete the transformation from college students to professionals. Fourth, it is suggested that the government should pay more attention to the group of college students exhibiting delayed employment, address the phenomenon of delayed employment in terms of employment situation propaganda, employment policy assistance, employment difficulties relief and job provision, and constantly alleviate the career decision-making difficulties faced by college students exhibiting delayed employment.

### 6.3. Limitations

Due to the limitations of individual research ability, this study was unable to explain the current career development problems faced by undergraduate students in China, who generally exhibit confusion, especially with respect to the formation of career goals. The postmodernist career self-management model focuses on the background, psychological and personality factors associated with individual variables, while the individual’s pluralistic value orientation and noncentral consciousness cause the evaluation criteria of college students to be vague. This study suggests that more empirical research methods should be used for further verification of these results.

## 7. Conclusion

During the COVID-19 epidemic, career decision-making difficulties among Chinese undergraduates became increasingly prominent and exhibited more diverse influencing factors and more complex generation mechanisms. Based on the theoretical framework of the career self-management model of SCCT theory, this study dynamically describes the factors influencing the career decision-making difficulties encountered by Chinese undergraduates, which not only include detailed individual factors but also focus on environmental factors against the backdrop of COVID-19. This study also summarizes the generation mechanism underlying both single-subject and multivariable career decision-making difficulties against the backdrop of many influencing factors. This study makes four practical contributions. First, the results of this study are helpful for delayed-employment undergraduates to understand their career decision-making and improve their career decision-making ability. Second, they are helpful for parents to understand the career psychology of undergraduates exhibiting delayed employment and to help their children make optimal career decisions. Third, this research provides a theoretical basis for employment services in colleges and universities and improves the pertinence and effectiveness of employment counseling. Fourth, it provides a theoretical reference for the improvement of the government’s vocational service system and promotes the full employment of Chinese graduates with higher quality.

## Data availability statement

The raw data supporting the conclusions of this article will be made available by the authors, without undue reservation.

## Ethics statement

The studies involving human participants were reviewed and approved by chuzhou university. The patients/participants provided their written informed consent to participate in this study.

## Author contributions

HS: conceptualization, writing—original draft, and writing—review and editing. The author has read and agreed to the published version of the manuscript.

## Funding

The study was supported by the following two projects: Anhui Provincial Department of Education Humanities and Social Sciences Key Project: Research on the Generation Logic and Acceleration Path of the “Slow Employment” Phenomenon of Local College Students (SK2020A0088); Anhui Province Ideological and Political Ability Improvement Project-Hairong Studio: Counselor Professional Ability Gas Station (sztsjh2019-2-36).

## Conflict of interest

The author declares that the research was conducted in the absence of any commercial or financial relationships that could be construed as a potential conflict of interest.

## Publisher’s note

All claims expressed in this article are solely those of the authors and do not necessarily represent those of their affiliated organizations, or those of the publisher, the editors and the reviewers. Any product that may be evaluated in this article, or claim that may be made by its manufacturer, is not guaranteed or endorsed by the publisher.

## References

[ref1] Bandura A. (1986). Social foundations of thought and action: A socio-cognitive theory. *NJ:Prentice* Hall: Englewood Cliffs.

[ref2] Brown S. D. Lent R. W. (1996). A social cognitive framework for career choice counseling. Career Dev. Q. 44, 354–366. doi: 10.1002/j.2161-0045.1996.tb00451.x

[ref3] Brown S. D. Lent R. W. (2019). Social cognitive career theory at 25: Progress in studying the domain satisfaction and career self-management models. J. Career Assess. 27, 563–578. doi: 10.1177/1069072719852736

[ref4] Chen X. (2000). Qualitative research in social sciences. Beijing: Educational Science Publishing House.

[ref5] Chen H. (2014). A review of research on career decision difficulties of college students in China. Chinese Vocational Tech. Educ. 36, 62–66.

[ref6] Chen S. Lu B. (2021). Peer influence:a new form of recessive ideological and political education. J. Xichang Univ.·Soc. Sci. Ed. 33, 24–27.

[ref7] Chen H. Pang L. Liu F. Fang T. Wen Y. (2022). “Be perfect in every respect”: the mediating role of career adaptability in the relationship between perfectionism and career decision-making difficulties of college students. BMC Psychol. 10:137. doi: 10.1186/s40359-022-00845-1, PMID: 35624459 PMC9145158

[ref8] Chen X. Yang C. Zhu K. (2022). Forecast of china’s economic growth rate for 2022 and policy suggestions. Bull. Chinese Acad. Sci. 37, 68–77.

[ref9] Chen S. P. Zhang Y. Wang X. (2012). The effects of implicit self-esteem and risk preference on college students’ career decision-making. J. Psychol. Sci. 35, 180–185.

[ref10] Chuang N. Lee P. C. Kwok L. (2020). Assisting students with career decision-making difficulties: can career decision-making self-efficacy and career decision-making profile help?, J. Hosp. Leis. Sport Tour. Educ. 26,:100235, doi: 10.1016/j.jhlste.2019.100235:100235

[ref11] Crites J. O. (1969). Vocational psychology. New York: McGraw-Hill.

[ref12] Darling N. Steinberg L. (1993). Parenting style as context: an integrative model. Psychol. Bull. 113, 487–496. doi: 10.1037/0033-2909.113.3.487

[ref13] Gadassi R. Gati I. Wagman-Rolnick H. (2013). The adaptability of career decision-making profiles: associations with self-efficacy, emotional difficulties, and decision status. J. Career Dev. 40, 490–507. doi: 10.1177/0894845312470027

[ref14] Gati I. Gadassi R. Saka N. Hadadi Y. Ansenberg N. Friedmann R.. (2011). Emotional and personality-related aspects of career decision-making difficulties: facets of career indecisiveness. J. Career Assess. 19, 3–20. doi: 10.1177/1069072710382525

[ref15] Gati I. Krausz M. Osipow S. H. (1996). A taxonomy of difficulties in career decision making. J. Couns. Psychol. 43, 510–526. doi: 10.1037/0022-0167.43.4.510

[ref16] Gati I. Saka N. (2001). High school Students' career-related decision-making difficulties. J. Couns. Dev. 79, 331–340. doi: 10.1002/j.1556-6676.2001.tb01978.x

[ref18] He Z. Zhou Y. Li F. Rao Z. Yang Y. (2021). The effect of proactive personality on college students’ career decision-making difficulties: moderating and mediating effects. J. Adult Dev. 28, 116–125. doi: 10.1007/s10804-020-09359-9

[ref19] Jepsen D. A. (1984). “The development perspective on vocational behavior:a review of theory and research” in Handbook of counseling psychology. ed. Brown S. D. (New York: Johns Willey-sons), 194–195.

[ref20] Jia Y. Hou Z. Zhang H. Xiao Y. (2022). Future time perspective, career adaptability, anxiety, and career decision-making difficulty: exploring mediations and moderations. J. Career Dev. 49, 282–296. doi: 10.1177/0894845320941922

[ref21] Karacan-Ozdemir N. (2019). Associations between career adaptability and career decision-making difficulties among Turkish high school students. Int. J. Educ. Vocat. Guid. 19, 475–495. doi: 10.1007/s10775-019-09389-0

[ref22] Kim S. Ahn T. Fouad N. (2016). Family influence on Korean students’ career decisions: a social cognitive perspective. J. Career Assess. 24, 513–526. doi: 10.1177/1069072715599403

[ref23] Konstantara M. K. Galanakis M. (2022). Organizational & industrial psychology in the 21<up>st</up> century—goal-setting theory and performance management: a systematic literature review. Psychology 13, 790–797. doi: 10.4236/psych.2022.135052

[ref24] Koumoundourou G. Tsaousis I. Kounenou K. (2011). Parental influences on Greek adolescents’ career decision-making difficulties: the mediating role of core self-evaluations. J. Career Assess. 19, 165–182. doi: 10.1177/1069072710385547

[ref25] Krumboltz J. D. (1993). Private rules in career decision making Columbus: National Center for Research in Vocation Education, Ohio State University.

[ref26] Laursen B. Veenstra R. (2021). Toward understanding the functions of peer influence: a summary and synthesis of recent empirical research. J. Res. Adolesc. 31, 889–907. doi: 10.1111/jora.12606, PMID: 34820944 PMC8630732

[ref27] Lent R. W. Brown S. D. (2006a). On conceptualizing and assessing social cognitive constructs in career research: a measurement guide. J. Career Assess. 14, 12–35. doi: 10.1177/1069072705281364

[ref28] Lent R. W. Brown S. D. (2006b). Integrating person and situation perspectives on work satisfaction: a social-cognitive view. J. Vocat. Behav. 69, 236–247. doi: 10.1016/j.jvb.2006.02.006

[ref29] Lent R. W. Brown S. D. (2019). Social cognitive career theory at 25: empirical status of the interest, choice, and performance models. J. Vocat. Behav. 115:103316. doi: 10.1016/j.jvb.2019.06.004

[ref30] Lent R. W. Brown S. D. Hackett G. (1994). Toward a unifying social cognitive theory of career and academic interest, choice, and performance. J. Vocat. Behav. 45, 79–122. doi: 10.1006/jvbe.1994.1027

[ref31] Lent R. W. Brown S. D. Hackett G. (2002). “Social cognitive career theory〔a〕.Brown D & Associates” in Career choice and development. 4th *Edn.* (San Francisco: Jossey-Bass).

[ref32] Lent R. W. Brown S. D. Tracey T. J. G. (2013). Social cognitive model of career self-management: toward a unifying view of adaptive career behavior across the life span. J. Couns. Psychol. 60, 557–568. doi: 10.1037/a0033446, PMID: 23815631

[ref33] Levitt H. M. Bamberg M. Creswell J. W. Frost D. M. Josselson R. Suárezorozco C. (2018). Journal article reporting standards for qualitative primary, qualitative meta-analytic, and mixed methods research in psychology: the APA Publications and communications board task force report. Am. Psychol. 73, 26–46. doi: 10.1037/amp0000151 29345485

[ref34] Li C. L. (2020). College graduate employment under the impact of COVID-19: employment pressure, psychological stress and employment choices. Educ. Res. 41, 4–16.

[ref35] Li X. Xiang G. Gui Y. (2021). Between materialism and post-materialism-changes in the job attitudes of graduates in post pandemic age. Beijing Cultural Review 75, 120–129.

[ref36] Liu B. Guo Y. Ao N. (2022). Impact of new COVID-19 pneumonia epidemic on employment quality of university graduates——comparative analysis based on the investigation of 19 universities before and after the epidemic. China Youth Study 320, 110–119.

[ref37] Liu Z. Zhang K. (2021). Review of researches on career decision-making difficulties. Contemporary Vocational Education 111, 66–78.

[ref38] Locke E. A. Latham G. P. (2006). New directions in goal-setting theory. Curr. Dir. Psychol. 15, 265–268. doi: 10.1111/j.1467-8721.2006.00449.x

[ref39] Long L. Peng Y. (2000). Research on foreign professional decision-making difficulties and its enlightenment. Ergonomics 6, 45–49.

[ref40] Lv Y. (2010). The relationship between career decision-making difficulties and parenting styles of college graduates. J. Henan Univ. (Soc. Sci.) 50, 142–146.

[ref41] Mao Y. Zhang Y. Bai J. Zhang L. Hu W. (2022). The impact of COVID-19 on the employment status and psychological expectations of college graduates: empirical evidence from the survey data of Chinese recruitment websites. Front. Psychol. 13:1039945. doi: 10.3389/fpsyg.2022.1039945 36438406 PMC9692114

[ref42] Martincin K. M. Stead G. B. (2015). Five-factor model and difficulties in career decision making: a Meta-analysis. J. Career Assess. 23, 3–19. doi: 10.1177/1069072714523081

[ref43] Ministry of Education. (2021). The number of college graduates in 2022 is estimated to be 10.76 million, an increase of 1.67 million over the same period last year. Available at: http://www.moe.gov.cn/fbh/live/2021/53931/mtbd/202112/t20211229_591046.html (Accessed July 28th).

[ref44] Nguyen M. H. La V. P. Le T. T. Vuong Q. H. (2022). Introduction to Bayesian Mindsponge framework analytics: an innovative method for social and psychological research. MethodsX 9:101808. doi: 10.1016/j.mex.2022.101808, PMID: 36034522 PMC9400117

[ref45] Nota L. Ferrai L. Solberg V. S. H. Soresi S. (2007). Career search self-efficacy, family support, and career indecision with Italian youth. J. Career Assess. 15, 181–193. doi: 10.1177/1069072706298019

[ref46] Palinkas L. A. Horwitz S. M. Green C. A. Wisdom J. P. Duan N. Hoagwood K. (2015). Purposeful sampling for qualitative data collection and analysis in mixed method implementation research. Admin. Pol. Ment. Health 42, 533–544. doi: 10.1007/s10488-013-0528-y, PMID: 24193818 PMC4012002

[ref47] Parola A. Marcionetti J. (2021). Career decision-making difficulties and life satisfaction: the role of career-related parental behaviors and career adaptability. J. Career Dev. 49, 831–845. doi: 10.1177/0894845321995571

[ref48] Sheu H. Bordon J. J. (2016). SCCT research in the international context. J. Career Assess. 25, 58–74. doi: 10.1177/1069072716657826

[ref49] Slaten C. D. Baskin T. W. (2014). Examining the impact of peer and family belongingness on the career decision-making difficulties of young adults: a path analytic approach. J. Career Assess. 22, 59–74. doi: 10.1177/1069072713487857

[ref50] Taylor K. M. (1982). An investigation of vocational indecision in college students: correlates and moderators. J. Vocat. Behav. 21, 318–329. doi: 10.1016/0001-8791(82)90040-9

[ref51] Taylor K. M. Betz N. E. (1983). Applications of self-efficacy theory to the understanding and treatment of career indecision. J. Vocat. Behav. 22, 63–81. doi: 10.1016/0001-8791(83)90006-4

[ref52] Tokar D. M. Withrow J. R. Hall R. J. Moradi B. (2002). Psychological separation, attachment security, vocational self-concept crystallization, and career indecision: a ructural equation analysis. J. Couns. Psychol. 50, 3–19. doi: 10.1037/0022-0167.50.1.3

[ref53] Vuong Q. Napier N. K. (2015). Acculturation and global Mindsponge: an emerging market perspective. Int. J. Intercult. Relat. 49, 354–367. doi: 10.1016/j.ijintrel.2015.06.003

[ref54] Wang D. Hou Z. Ni J. Tian L. Zhang X. Chi H.. (2020). The effect of perfectionism on career adaptability and career decision-making difficulties. J. Career Dev. 47, 469–483. doi: 10.1177/0894845318803192

[ref55] Ye L. Ding W. Ning Z. Ting L. (2022). The influencing factor and coping strategy for the college students' career decision-making in the post-epidemic period. Future Dev. 46, 28–35.

[ref56] Yin R. K. (2014). Case study research: Design and methodology (5th *Edn.*). London, Sage Publications.

[ref57] Yue C. Qiu W. (2022). College Graduates' employment and influencing factors in the context of the regular COVID-19 pandemic prevention and control. Educ. Res. 43, 28–44.

[ref58] Zhang S. Ding L. Hu D. Si J. (2016). Relationship between self-supporting personality and career decision-making difficulties in college students: a mediating role of state-trait anxiety. Chinese. J. Clin. Psychol.

[ref59] Zhao H. (2012). A study on the influencing factors of college Students' career decision-making difficulties. Hubei Soc. Sci. 309, 160–163.

[ref60] Zhao H. (2013). On relationship between family factors and career decision-marking difficulties of college students. J. Jixi University 13, 31–32.

[ref61] Zheng X. Wang D. (2019). The causes and countermeasures of "delayed employment" of college graduates. Soc. Sci. Front. 285, 276–280.

[ref62] Zhou R. (2022). Contemporary college students’ employment mentality: the new Normal and its countermeasures from the perspective of social ecology. Contemp. Youth Res. 377, 94–101.

